# Supplementary material for: Circular wood use can accelerate global decarbonisation but requires cross-sectoral coordination
Source: Nat Commun. 2023 Oct 25;14:6766. doi: 10.1038/s41467-023-42499-6 (PMC10600095; doi:10.1038/s41467-023-42499-6)
Supplement: Supplementary file 1 — Supplementary Information [file 41467_2023_42499_MOESM1_ESM.pdf]

Supplementary Information

Circular wood use can accelerate global decarbonisation but requires cross-sectoral coordination

Eilidh J. Forster*[1], John R. Healey[1], Gary Newman[2], & David Styles[1,3]
[1] School of Natural Sciences, Bangor University, Bangor, Gwynedd, LL57 2UW, UK
[2] Woodknowledge Wales Ltd., Ffarm Moelyci, Felin Hen Road, Tregarth, Gwynedd, LL57 4BB, UK
[3] School of Biological & Chemical Sciences and Ryan Institute, University of Galway, Galway, H91 TK33, Ireland

Supplementary Table 1 - Life Cycle Inventory for ‘business as usual’ (BAU) wood use

Inventory of key inputs and outputs for processes considered along the life cycle of forestry value chains derived from thinned forest systems over 100 years. Emissions factors (EF) and their sources are indicated. GWP is global warming potential (measured in kg CO_2e).

Process stage	Input/output/process	Activity data source	Units	Thinned		EFs	EF source
				In	Out	GWP	
Planting (1&2)	Tree seedlings	GH[2]	Item(s)	774,012,298		0	Ecoinvent[1]
	15 tonne 360 Excavator	GH[2]	hrs	464,407		65	Ecoinvent[1]
	Pesticides (acetamiprip)	Industry recommended	kg	25,759			Ecoinvent[1]
Forest management	Harvester (diesel use)	GH[2]	hrs	1,207.459		56	Ecoinvent[1]
	Forwarder (diesel use)	GH[2]	hrs	1,207,459		46	Ecoinvent[1]
	Harvested wood	CBM-CFS3[3]	m^3		13,751,783		IPCC
Transport (forest to processor)	>32 t truck, EURO6	GH[2]	t.km	1,909,601,142			Ecoinvent[1]
Debarking	Harvested wood	CBM[3], GH[2]	m^3	11,634,008			
	Diesel	Ecoinvent[1]	MJ	10.250,052			
	Lubricating oil	Ecoinvent[1]	kg	9,095			
	Bark chips	GH[2], FR CFs[5]	kg		865,458,517	20	Ecoinvent[1]
	Debarked wood	GH[2], FR CFs[5]	m^3		10,387,507		
Sawing	Diesel (internal transport)	Ecoinvent[1]	MJ	105,021,592			
	Electricity	Ecoinvent[1]	kWh	70,229,933			
	Lubricating oil	Ecoinvent[1]	kg	382,034			
	Debarked wood	GH[2], FR CFs[5]	m^3	7,305,635			
	Sawnwood	JJ&S[4]	m^3		4,018,099	25	Ecoinvent[1]
	Sawmill residues	JJ&S[4]	kg		1,141,285,057		
Drying (of sawn timber)	Electricity	Ecoinvent[1]	kWh	67,102,253			
	Sawnwood	JJ&S[4]	m^3	4,018,099			
	Sawnwood - dried (u=20%)	Assume no loss in volume during drying	m^3		4,018,099	29	Ecoinvent[1]
Planing	Electricity	Ecoinvent[1]	kWh	34,840,025			
	Sawnwood (carcassing) dried (u=20%)	JJ&S[4]	m^3	4,018,099			
	Sawnwood (carcassing) planed	Vol loss accounted for in 'sawing'	m^3		4,018,099	35	Ecoinvent[1]
	Sawmill residues	JJ&S[4]	kg		1,141,285,057		
Chemical treatment	Electricity	Ecoinvent[1]	kWh	708,223			
	Wood preservative	Ecoinvent[1]	kg	991,511,510			
	Sawnwood (fencing) dried (u=20%)	JJ&S[4]	kg	688,296,981			
	Debarked wood (fence poles)	GH[2],FR CFs[5]	kg	303,214,529			
	Preserved wood	No vol. change	kg		991,511,510	0	Ecoinvent[1]
Particle board production	Electricity	Ecoinvent[1]	kWh	345,191,890			
	Heat	Ecoinvent[1]	MJ	4,050,362,146			
	Resin	Ecoinvent[1]	kg	169,276,792			
	Debarked wood (chip)	GH[2]	kg	596,750,357			
	Sawmill residues	JJ&S[4]	kg	700,595,575			
	Recycled wood	FC report	kg	1,108,766			
	Particle board	FR CFs[5]	m^3		3,415,000	262	Ecoinvent[1]
Fibre board production	Electricity	Ecoinvent[1]	kWh	685,822,000			
	Heat	Ecoinvent[1]	MJ	4,757,462,000			
	Debarked wood (chip)	GH[2] FR CFs[9]	kg	596,750,357			
	Sawmill residues	JJ&S[4]	kg	647,847,106			
	Fibre board	JJ&S[4], GH[2], FR CFs[5]	m^3		1,370,000	98	Ecoinvent[1]
Woodchip production (for biomass energy)	Electricity	Ecoinvent[1]	kWh	71,358,404			
	Lubricating oil	Ecoinvent[1]	kg	5,642			
	Harvested wood - 'fuel'	GH[2]	kg	765,829,625			
	Recycled wood - 'biomass'	FC	kg	2,000,000,000			
	Wood chips	GH[2]	kg, dry		2,765,829,625	0	Ecoinvent[1]
Biomass energy	Electricity	Ecoinvent[1]	kWh		20,284,594,466		Ecoinvent[1]
	Wood chips	GH[2]	Kg, dry	2,765,829,625			Conversion biogenic C to CO_2 eq
	Heat	Ecoinvent[1]	MJ		52,550,762,866	0	Ecoinvent

Process stage	Input/output/process	Activity data source	Units	Thinned		EFs	EF source
				In	Out	GWP	
Avoided fossil fuels	Electricity generation (natural gas, high pressure)	Ecoinvent[1]	m^3	3,146,931,268			Ecoinvent[1]
Avoided construction materials	140 mm concrete block and mortar wall replaced by timber frame wall	BRE[6]	m^2	18,779		37	Ecoinvent[1]

Supplementary Methods 1 - Wood use scenarios

Wood use scenarios assessed in the present study

Circular use:

To match the research objectives of the paper, we selected recycled MDF as the circular case study because it is an almost closed-loop process (hence it is a circular, rather than cascading, use). It was also determined to be a suitable scenario for this study because it applies proven technology to produce (a recycled version of) an already established product with a known market size in the UK; and the collection of waste wood (including MDF) is already common practice in the UK. Furthermore, we were able to obtain sufficient data to meet the requirements for rigorous LCA, specifically:

1) the annual volume of recovered MDF at the UK national scale of our study
2) the volume of MDF currently manufactured in the UK
3) lifecycle inventory data for the process (which uses proven technology that is being commercially scaled-up)

Cascading use:

The UK value chain also presents opportunities for improved hierarchical cascading use. The major feature of the cascading wood use case selected for the present study is enhanced hierarchical use, which inherently leads to a higher degree of cascading use (i.e. sequential use of wood in multiple applications). In the UK there is very little landfilling of waste wood, and the majority of recovered waste wood goes into cascading uses: primarily particle board manufacturing (which has 50% recycled wood content in UK) and bioenergy. By increasing the volume of sawnwood produced, the future volume of recovered waste wood (sawnwood) cascading to other uses (in our example particle board manufacturing) also increases.

There are significant additional opportunities for enhancing and adding further levels of cascading use to the UK wood value chain. However, our selection of pathways and scenarios for analysis was governed by the stringent data requirements of the rigorous LCA methodology that we have utilised. We carefully surveyed a range of other existing pathways and scenarios but due to their complexity and major gaps in available data, none of them met the criteria of being suitable for rigorous LCA. This limitation was particularly acute for emerging technologies. This represents a conservative approach, given that various prospective sequential uses (examples below) could further increase the extent of, and GWP credit derived from, cascading and/or circular value chains in future.

Alternative wood use scenarios

There are many other promising cascading and circular economy value chain alternatives, at different stages of development and application that could be derived from wood. These include:

- Bioplastics/biocomposites – these can replace many types of petrochemical plastics and can be recyclable

- Biobased chemicals – these can have multiple applications (with varying product substitution impacts), including: biobased fragrances and flavours; lubricants; resins; cleaners and solvents

- Biobased materials, such as microfibrillated cellulose (MFC), carbon fibre and binders – these have a wide range of potential applications including packaging, textiles, energy storage (batteries) and construction products

- Advanced second generation biofuels - biofuels manufactured from wood can replace fossil fuels[7].

- Biochar - there is a small established market for biochar produced from pyrolysis of wood or other biomass, which can be used as a soil improver[8] and for many other purposes[9]. Application of biochar to the certain soils can store the contained carbon and keep it out of the atmosphere for many decades. Many more emerging applications of biochar are being developed, including addition to concrete to create a long-term carbon sink[10].

- Mass timber, based on engineered wood products such as cross-laminated timber (CLT), dowel-laminated timber (DLT) or glue-laminated timber (glulam or GLT), manufactured from virgin wood, comprising multiple layers of solid wood – these are used to replace concrete and steel or conventional sawnwood beams in construction.

Production of these materials overwhelmingly uses virgin wood rather than recovered waste wood as the primary feedstock, at present. It is possible that in the future new processes will be developed enabling the utilisation of recovered waste wood. A specific promising example is cross-laminated secondary timber (CLST), which utilises recovered waste wood (e.g. recovered floorboards), and is a good example of cascading wood use. However, such a value chain remains uncertain and challenging to implement (and model) as technical barriers remain to be overcome, such as definition of the minimum level of quality and size of recovered sawnwood (strength grading is more complex with reclaimed timber). Even with more certainty on the specification of sawnwood eligible for CLST production, the collection and reporting of data on the quantity and quality of recovered sawnwood in the UK is insufficient to derive robust national scenarios – particularly where consequential GWP impacts of any changes would require information on how that wood is currently being cascaded.

In essence, there is a huge range of options for circular and cascading wood use that should be included in more prospective assessment of wood value chains in order to understand the full climate mitigation potential of wood. The examples mentioned above have limited availability (often due to commercial sensitivities) of the process data required to perform LCA (as listed in points 1 to 3 above). The products also have different qualities with respect to recoverability, recyclability and cascading use; and many will have considerably different product life durations, all with significant implications for lifecycle GWP impact. The scale of each of these product markets is also very uncertain, which will also have important implications on net demand and competition for (limited)

resources, with complex consequential GWP impacts. Further study is urgently needed to assess these options as technical data emerges from pilot studies; in the meantime, the exclusion of these commercially unproven products is a conservative approach in this study with respect to our conclusions.

Supplementary Methods 2 - Decarbonisation Assumptions

Decarbonisation of the forestry value chain is highly dependent on the development of zero emissions mobile machinery and heavy goods vehicles (HGVs), as well as electricity and gas grid decarbonisation. A summary of the decarbonisation projections used in the study are summarised in Table 1 and described below. Calculations are also contained in 'Supplementary_Data_1_(BAU_LCA)'.

Table 1 – Industrial decarbonisation projection assumptions for greenhouse gas (GHG) emissions from forestry operations, wood processing and transport, and from production of substituted products (concrete and fossil fuels). For clarity, 100% indicates zero GHG emissions.

	**Time period**				**Source**
**Sub-sector**	2020-2029	2030-2039	2040-2049	2050-2059	
Forestry operations	13%	33%	67%	100%	CCC Sixth Carbon budget report[11]
Sawmills & woodfuel production	39%	62%	82%	92%	(Wood fuel assumed to be similar to sawmills).
Wood panel production	25%	60%	70%	79%	SLR Consulting Ltd.[12]
**Energy supply**					
Gas grid	5%	10%	20%	30%	SLR Consulting Ltd[12]
Electricity grid	50%	77%	95%	100%	CCC Sixth Carbon budget report[12]
**Substitution credits**					
Concrete production	5%	10%	20%	33%	WSP Global Inc. & DNV GL[13]

Forestry operations
Projections for forestry operations are taken from the Committee for Climate Change Sixth Carbon Budget[1] 'balanced net zero pathway' for decarbonising heavy goods vehicles (HGVs) (optimistically, given the remote and arduous nature of operations); agricultural machinery has similar decarbonisation projections in the CCC report.

Sawmills & wood fuel production

Projections for decarbonising sawmills are calculated using the projections for forestry operations (mobile plant), the electricity grid and gas grid; and apportioned according to the breakdown of energy sources and tonnage (averaged across the sector) from primary UK sawmill data (2020)[14].

Woodfuel production decarbonisation is assumed to be similar to sawmills since the process is also highly electrified and employs mobile plant for moving material around site.

Wood panel production
The Wood Panel Industry Federation (WPIF) commissioned a report[12] (by SLR Consulting Ltd) in 2022 to outline a decarbonisation pathway for the wood panel industry in the UK and Ireland. The

‘realistic’ decarbonisation pathway proposed in this report is used to project decarbonisation of the wood panel production in this study.

Electricity grid
The projection for electricity grid decarbonisation is taken from the Committee for Climate Change Sixth Carbon Budget ‘balanced net zero pathway’ for electricity generation[12].

Gas grid
Gas decarbonisation is highly uncertain and will be regionally dependent[11,15] (CCC report and National grid report). Therefore, we use the assumptions proposed in the WPIF report regarding gas decarbonisation, which takes into account industry specific barriers, including remote location.

Concrete production
The decarbonisation projection used for cement production is the ‘maximum technical pathway – without carbon capture and storage’ for decarbonisation of cement production, taken from the ‘Industrial Decarbonisation & Energy Efficiency Roadmaps to 2050 – Cement’ report, prepared in 2015 for the UK government Department of Energy and Climate Change and the Department for Business, Innovation and Skills[13]. This pathway assumes decarbonisation of 33% by 2050.

Supplementary References

1. Wernet, G. et al. The ecoinvent database version 3 (part I): overview and methodology. *Int. J. Life Cycle Assess*. **21**, 1218–1230 (2016).
2. Gresham House. Forest production data. (2018).
3. Kull, S. J., Northern Forestry Centre (Canada) & Canada. Natural Resources Canada. Operational-scale carbon budget model of the Canadian forest sector (CBM-CFS3) : version 1.2, user's guide. (2016).
4. James Jones & Sons. Sawmill production data provided from personal correspondence. (2019).
5. Matthews, R. W., Jenkins, T. A. R., Mackie, E. D. & Dick, E. C. Forest Yield: A handbook on forest growth and yield tables for British forestry. (2016).
6. BRE. IMPACT database v5 (accessed via etool LCA software). (2018).
7. Lee, R. A. & Lavoie, J.-M. From first- to third-generation biofuels: Challenges of producing a commodity from a biomass of increasing complexity. *Animal Frontiers* **3:2**, 6–11 (2013). https://doi.org/10.2527/af.2013-0010
8. Woolf, D. et al. Sustainable biochar to mitigate global climate change. *Nat Commun* **1**, 56 (2010). https://doi.org/10.1038/ncomms1053
9. Bolan, N. et al. Multifunctional applications of biochar beyond carbon storage. International *Materials Reviews* **67**, 150–200 (2022). https://doi.org/10.1080/09506608.2021.1922047
10. Suarez-Riera, D., Restuccia, L. & Ferro, G. A. The use of Biochar to reduce the carbon footprint of cement-based materials. *Procedia Structural Integrity* **26**, 199-210 (2020). https://doi.org/10.1016/j.prostr.2020.06.023
11. Committee on Climate Change. The Sixth Carbon Budget - The UK's path to Net Zero. Presented to the Secretary of State pursuant to section 34 of the Climate Change Act 2008. (2020)
12. SLR Consulting Ltd. Decarbonisation of UK and Ireland wood panel manufacturing sector. Prepared for Wood Panel Industries Federation. (2022)
13. WSP Global Inc. Industrial Decarbonisation & Energy Efficiency Roadmaps to 2050 – Cement. Prepared for UK Department of Energy and Climate Change and the Department of Business, Innovation and Skills (2015)
14. UK Sawmill primary data. Quantity and source of consumed energy for 20 UK sawmills. Provided confidentially. (2020)
15. National Grid Electricity System Operator Limited. Future Energy Scenarios. Strategic Insights (2019)
