# Supplementary material for: Circular wood use can accelerate global decarbonisation but requires cross-sectoral coordination
Source: Nat Commun. 2023 Oct 25;14:6766. doi: 10.1038/s41467-023-42499-6 (PMC10600095; doi:10.1038/s41467-023-42499-6)
Supplement: Supplementary file 3 — Description of Additional Supplementary Files Document [file 41467_2023_42499_MOESM3_ESM.pdf]

Description of Additional Supplementary Files

Supplementary Data Legends:

Supplementary_Data_1 (BAU_LCA) -

Lifecycle assessment (LCA) inventory and impact calculation file for business as usual (BAU) wooduse value chain scenario.

Supplementary_Data_2 (casc_LCA) -

Lifecycle assessment (LCA) inventory and impact calculation file for enhanced cascading (casc) wooduse value chain scenario.

Supplementary_Data_3 (circ_LCA) -

Lifecycle assessment (LCA) inventory and impact calculation file for the enhanced circular (circ) wood-use value chain scenario.

Supplementary_Data_4 (casc&circ_LCA) -

Lifecycle assessment (LCA) inventory and impact calculation file for the enhanced cascading and circular (casc&circ) wood-use value chain scenario.
